# Performance Evaluation of Phenol-Resin-Based Adsorbents for Heat Transformation Applications

**DOI:** 10.3390/ma16155262

**Published:** 2023-07-26

**Authors:** Hafiz M. Asfahan, Muhammad Sultan, Muhammad Farooq, Fahid Riaz, Sobhy M. Ibrahim, Md Shamim Ahamed, Muhammad Imran

**Affiliations:** 1 Department of Agricultural Engineering, Bahauddin Zakariya University, Multan 60800, Pakistan; hmasfahan@gmail.com; 2 Department of Mechanical Engineering, University of Engineering and Technology, Lahore 39161, Pakistan; engr.farooq@uet.edu.pk; 3 Mechanical Engineering Department, Abu Dhabi University, Abu Dhabi P.O. Box 59911, United Arab Emirates; 4 Department of Biochemistry, College of Science, King Saud University, P.O. Box 2455, Riyadh 11451, Saudi Arabia; syakout@ksu.edu.sa; 5 Department of Biological and Agricultural Engineering, University of California, Davis, CA 95616, USA; mahamed@ucdavis.edu; 6 Department of Mechanical, Biomedical and Design Engineering, College of Engineering and Physical Sciences, Aston University, Birmingham B4 7ET, UK; m.imran12@aston.ac.uk

**Keywords:** phenol resins, ideal cycle, specific cooling energy, specific heating energy

## Abstract

Phenol resins (PRs) are considered as relatively inexpensive adsorbents synthesized from agricultural biomass via employing a variety of synthesized procedures. The performance of PR for heat transformation application is not widely investigated. In this regard, the present study aims to evaluate the four PR derivative/refrigerant pairs, namely (i) KOH6-PR/CO_2_, (ii) SAC-2/HFC, (iii) KOH4-PR/ethanol, and (iv) KOH6-PR/ethanol, for adsorption cooling and adsorption heating applications. Ideal cycle analyses and/or thermodynamic modelling approaches were utilized comprising governing heat and mass balance equations and adsorption equilibrium models. The performance of the AHP system is explored by means of specific cooling energy (SCE), specific heating energy (SHE), and coefficient of performance (COP), both for cooling and heating applications, respectively. It has been realized that KOH6-PR/ethanol could produce a maximum SCE of 1080 kJ/kg/cycle and SHE of 2141 kJ/kg/cycle at a regeneration temperature (T_reg_) and condenser temperature (T_cond_) of 80 °C, and 10 °C, respectively, followed by KOH4-PR/ethanol, SAC-2/HFC-32, and KOH6-PR/CO_2_. The maximum COP values were estimated to be 1.78 for heating and 0.80 for cooling applications, respectively, at T_reg_ = 80 °C and T_cond_ = 10 °C. In addition, the study reveals that, corresponding to increase/decrease in condenser/evaporator pressure, both SCE and SHE decrease/increase, respectively; however, this varies in magnitude due to adsorption equilibrium of the studied PR derivative/refrigerant pairs.

## 1. Introduction

Research activities are shifting toward the development of energy-efficient solid sorption, namely adsorption heat pump (AHP) systems, which are acknowledged as a credible route for minimizing the exponential growth in energy demand in the air conditioning (AC) sector. Mechanical vapor compression (MVC) AC systems have a high coefficient of performance (COP) ranging between 3.0 and 5.0, consequently indicating their wide implementation as a convenient commercialized cooling/heating system [1,2]. However, environmental consequences aligned with the MVC-AC system include high global warming potential, emission of harmful greenhouse gasses, destruction of the ozone layer, and prominently alleviating the paucity of natural fossil fuel reserves [3]. On the other hand, radiative cooling technology that utilizes the principles of thermal radiation to achieve cooling without the need for mechanical compression holds great promise [4]. However, radiative cooling requires clear sky conditions and the need for direct exposure to the sky for effective heat dissipation [4]. The AHP system scavenges discarded low-grade waste heat, abundantly available in the surrounding environment, as a prime mover, thereby contributing its share to mitigating the environmental pollution [1,5].

At the very core of an AHP system, the adsorbent is considered as the vital driving entity that directly influences the performance of the AHP system [6,7]. The adsorbent material captures the refrigerant vapors based on their active free sites, surface area, pore volume, and even on the refrigerant flow patterns [8]. The possible choice for the AHP system is an adsorbent material having hygroscopic characteristics [9,10]. In this regard, various adsorbents belonging to different classes were investigated in the past [11,12]. For instance, silica gels (SGs) are a commonly employed adsorbent class in the AHP system. Typically, the surface area of SG ranges between 586.0 and 863.6 m^2^/g, having a pore volume of 0.41–0.489 cm^3^/g [13,14]. The studies relevant to SG/water pair are presented here for completeness. The potential of an SG/water pair for adsorption cooling (AC) was explored and it was found that specific cooling energy (SCE) of 176 W/kg was measured at 80 °C regeneration temperature (T_reg_) with the COP of 0.45 [15]. Similarly, an experimental facility of adsorption chiller is developed in the literature comprising an SG/water pair [16]. The SCE and COP were recorded to be 104.60 W/kg and 0.39, respectively, at 80 °C of T_reg_. Peter et al. [17] mathematically investigated the performance of an SG/water pair by configuring four adsorption beds. It was reported that an SCE of 15.0 R-ton/ton could be produced. Wang et al., [18] developed a lumped numerical model, which reported that an SCE of 5.0 R-ton/ton at 85 °C T_reg_ can be obtained from an SG/water pair. In the same manner, for adsorption heating (AH), J. Pinheiro et al. [19] realized a specific heating energy (SHE) of 802 J/kg with a COP of 1.02 by employing an SG/water pair.

Activated carbons (AC) are another adsorbent class highly investigated in communication with different refrigerants [20]. Zhao et al. [21] studied the AC/methanol combination and observed a COP of 0.11 at 110 °C T_reg_. Another study reveals that the AC/methanol pair produced an SCE of 16 W/kg and entailed a COP of 0.125 when Treg was set at 120 °C [22]. El-Sharkawy et al. [23] experimented with methanol adsorption on Maxsorb III and performed isotherm modeling using the Dubinin–Raduskevich (D–R) model. Additionally, a thermodynamic model was utilized to explore the cooling potentials obtained from Maxsorb III/methanol pairs. The results showed that an SCE of 448.0 W/kg with a COP of 0.77 could be obtained; however, if compared with the activated charcoal/methanol pair, the SCE recorded 731 W/kg at T_reg_ = 90 °C and T_evap_ = 7 °C. If T_evap_ reduces to –5 °C, the SCE and COP are significantly decreased. Similarly, the performance of AC/ethanol is also reported for the AC system. For instance, it has been reported that Maxsorb-III/ethanol can produce 420 kJ/kg SCE at an T_evap_ of 7 °C and T_reg_ 80 °C [24]. One can refer to the cited articles for exploring the performance of activated carbon/ethanol pairs [25,26,27]. The AC/ammonia pair was also investigated for the AC system. For instance, a laboratory-scale refrigerator was developed, operating at T_evap_ of –1 °C. Similarly, a simple test rig was developed, driving with hot and cold airstreams for desorption and adsorption of ammonia from the AC [28]. It was identified that the system is capable of producing an SCE of 400 W/kg with COP 0.85.

Metal–organic frameworks (MOFs) are the emerging adsorbent class highly investigated for AHP systems due to their tremendous tunable porous structure, high surface area, and adsorption uptake. For instance, CPO-27(Ni) has a surface area of 1113–1337 m^2^/g with a pore volume of 0.39–0.54 cm^3^/g [29,30] when investigated for the AC system, and the SCE of 216 R-ton/ton was recorded [31]. MIL-101 has a surface area of 4000 m^2^/g [32] with a pore volume of 1.51 cm^3^/g [33] and can produce an SCE of 89.7 R-ton/ton [34]. Similarly, aluminum fumarate can produce an SCE of 185 W/kg at T_reg_ = 80 °C [35]. Adsorption of other refrigerants, such as CO_2_ adsorption on MOFs, is also investigated in the literature [36,37]. However, the MOFs are expensive, have complex synthesis procedures, deterioration over time, and low hydro-thermal stability, hindering their wider implementation. Significant research efforts are directed towards the advancement of MOFs, with a particular focus on enhancing their adsorption capabilities and optimizing their thermophysical properties [38,39]. A low-cost sustainable adsorbent is principally required to develop an energy-efficient AHP system. 

Phenol resin (PR) is another adsorbent class manufactured from agriculture biomass for various heat transformation applications. El-Sharkawy et al. [40] developed two PR derivatives activated with potassium hydroxide (KOH), namely KOH4-PR and KOH6-PR, in communication with ethanol as a refrigerant from the perspective of developing a next-generation AC system. In this regard, the isothermal characterization was performed using thermogravimetric analysis. In addition, adsorption isotherm modeling was performed using a Dubinin–Astakhov (D–A) model. The results indicated that the KOH6-PR is capable of uptake of 2 kg of ethanol per kg of KOH6-PR and 1.43 kg of ethanol per kg of KOH4-PR. Uddin et al. [41] investigated CO_2_ adsorption on KOH6-PR. The results reveal that the KOH6-PR ethanol can uptake 1.69 kg of CO_2_ per kg of KOH6-PR. Sultan et al. [42] investigated the adsorption of HFC-32 on spherical activated carbon (SAC-2) and reported uptake of 2.34 kg/kg. These PR derivative/refrigerant pairs could outperform if employed in the AHP system due to their porosity and adsorption potentials.

In this realm, the present study aims to investigate the performance of four kinds of phenol resin (PR) derivative/refrigerant pairs abbreviated as (i) KOH6-PR/CO_2_, (ii) SAC-2/HFC-32, (iii) KOH4-PR/ethanol, and KOH6-PR/ethanol for an AHP system that mutually exhibited adsorption cooling and adsorption heating applications. To the best of the author’s knowledge, the selected PR derivative/refrigerant pairs have not been investigated for twin applications. The thermophysical properties and fitting constants of the adsorption isotherm model for the selected PR derivative/refrigerant pairs are available via [40,41,42] and utilized accordingly in the present research. A steady-state thermodynamic modeling scheme is utilized comprising the Dubinin–Astakhov model, and the system’s governing heat and mass balance equations are widely accessible and often employed in the literature [43,44]. The key performance indicators of the AHP system, including specific cooling energy (SCE), specific heating energy (SHE), and COP, are evaluated at steady-state conditions. Furthermore, the study also performs a sensitivity analysis in order to explore the effect of regeneration temperature (T_reg_), adsorption temperature (T_ads_), condenser pressure (P_cond_), and evaporator pressures (P_evap_) on the studied key performance indicators of the AHP system. Overall, the study contributes positively regarding the selection of the potential PR derivative/refrigerant pair for the AHP system.

## 2. Adsorption Heat Pump (AHP)

**Figure 1:**
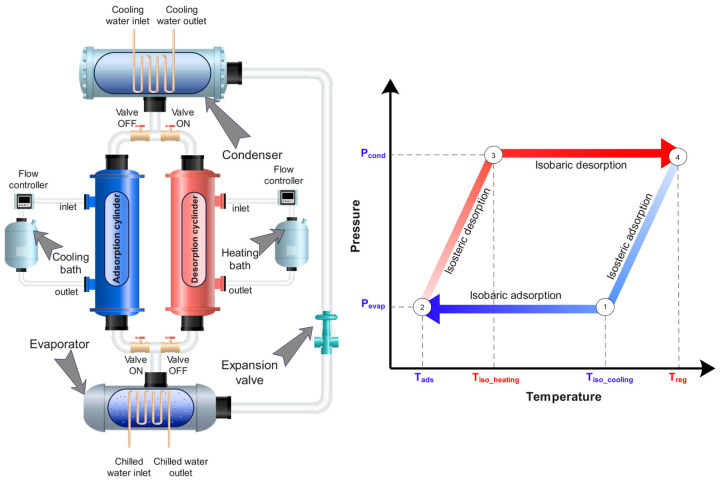
Working schematic of the AHP system (**left**) and ideal cycle pressure–temperature–uptake diagram (**right**).

The working of AHP follows a close cyclic configuration, employing well-known thermodynamic processes, i.e., adsorption, isosteric heating, regeneration, and isosteric cooling, respectively. Generally, the AHP system contains an expansion valve, heating/cooling water baths, and four cylinders retrofitted with heat exchangers. Two cylinders within the AHP device are packed with porous adsorbent material, while the remaining two cylinders serve as an evaporator and condenser, respectively. Indeed, the performance of the AHP system relies heavily on the type of adsorbent material, associated adsorption equilibrium, as well as thermophysical properties. The working schematic and ideal cycle of the AHP system are showcased in Figure 1 (left) and (right), respectively. The thermodynamic processes are accomplished in the cylinders packed with the porous adsorbent material; however, they have been controlled/regulated with the externally connected heating/cooling water baths in order to maintain the required thermal fronts. For instance, to facilitate the adsorption process, a cooling bath is connected from the perspective of harnessing adsorption heat released during the process, thereby augmenting the overall adsorption capacity. During the adsorption process, the refrigerant undergoes a transition from liquid to vapor within the evaporator via utilizing surrounding heat. Subsequently, the vapor is effectively trapped within the pore spaces of the adsorbent material, facilitated by the establishment of Van der Waals forces of interaction and driven by significant affinity between the refrigerant and the adsorbent. As the adsorption process is performed at constant evaporator pressure, it is thereby termed as isobaric adsorption. Similarly, for enabling the desorption process, an external heating source is required (i.e., heating bath), causing the breakdown of the Van der Waals force of attraction. As a result, the refrigerant molecules are liberated from the adsorbent pore spaces under conditions of high temperature and pressure and subsequently proceed towards the condenser. The coolant circulating inside the condenser heat exchanger captures the latent heat of the refrigerant and supports the refrigerant phase transition. A high-pressure liquid with relatively low-temperature refrigerant is produced, which is then directed to the expansion valve. The expansion valve undergoes the refrigerant to experience a sudden drop in pressure, leading to a corresponding decrease in temperature. As a result, a low-pressure liquid–vapor mixture is generated, which is then directed to the evaporator to initiate the subsequent cycle. Prior to the adsorption and desorption processes, isosteric cooling and isosteric heating processes are conducted with the aim of adjusting the cylinder pressure to match the pressure of the condenser and evaporator, respectively. Both processes, also known as switching times, allow the cylinder to prepare itself for the subsequent adsorption and desorption processes by increasing or decreasing the pressure accordingly. The isosteric heating temperature (T_iso_heating_) and isosteric cooling temperature (T_iso_cooling_) are the intermediate temperatures values beyond which the desorption and adsorption processes are executed, respectively. The extended details relevant to the determination of these temperature points are provided in Section 4.

The AHP device can potentially be used both for both cooling and heating applications. The cooling potential (Q_evap_) is realized in the AHP cycle during the evaporation of refrigerant in the evaporator, whereas the heating potential is realized during condensation (Q_cond_) and adsorption (Q_ads_) processes. The steady-state operating conditions of the AHP system used for the present study are summarized in Table 1.

## 3. Materials

In this study, four kinds of PR derivative/refrigerant pairs, i.e., (i) KOH6-PR/CO_2_ [41], (ii) SAC-2/HFC-32 [42], (iii) KOH4-PR/ethanol [40], and (iv) KOH6-PR/ethanol [40], are studied from the viewpoint of thermodynamically analyzing their performances for AHP system exhibiting both adsorption cooling and heating applications. The comprehensive discussion and procedure regarding the manufacturing as well as thermophysical features and structures of the PR derivatives can be found from the cited literature [40,41,42]. 

**Figure 2:**
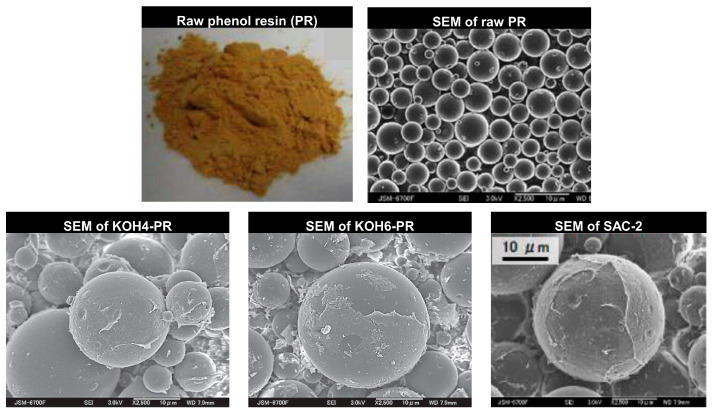
Pictorial and SEM images of studied raw PR and associated investigated for the AHP system, reproduced with permission from [40,41,42].

A quick overview regarding the synthesizing of the studied PR derivatives in order to encompass all facets is as follows [44,45,46]: PR derivatives were prepared via carbonization of the raw PR occurring at 600 °C for an hour at a heating rate of 5 °C/min in the presence of nitrogen (N_2_) flow. For activation, KOH was utilized as activating agent. Two weight ratios (KOH/carbonized PR) of 4 and 6 were subjected to heat treatment process at a heating rate of 5 °C/min and maintained at 900 °C for one hour under N_2_ flow. To eliminate the salts generated during the heat treatment and residual KOH, the samples underwent a rigorous washing procedure. This involved subjecting them to multiple rinses with a HCl solution and a subsequent rinse with deionized water for a pH of 7. The collected sample was subjected to two-step drying process. Primary drying takes place at 100 °C for three hours while secondary drying takes place at 150 °C for 12 h. Figure 2 shows the pictorial of raw PR, entailing SEM images of raw PR and associated derivatives. The porous properties, elemental composition, and thermal properties of the PR derivatives are presented in Table 2 [40,41,42].

A thermodynamic model based on the adsorption equilibrium model and governing heat and mass balance equations are utilized and/or programmed in Python from the perspective of investigating the PR derivative/refrigerant pairs for the AHP system. The developed Python program is capable of estimating the heating and cooling potentials of AHP corresponding to different operating conditions (i.e., regeneration and adsorption temperature). In addition, the effect of evaporator and condenser pressure is analyzed for each PR derivative/refrigerant pair. The subsequent subsections entail the specifics of the adsorption equilibrium model as well as a comprehensive discussion regarding the thermodynamic equations and Python libraries used in this study.

### 3.1. Adsorption Equilibrium

Dubinin–Astakhov (D–A) isotherm model is used for simulation of the adsorption isotherms. The well-optimized fitting constants for the D–A model corresponding to each PR derivative/refrigerant pair are provided in Table 3 and available via [40,41,42]. The D–A isotherm model is presented in Equation (1) as provided by [46]:

(1) $$w=w_{o}\exp\left[-{\left(\frac{R_{o}T}{E}\ln\frac{P_{s}}{P}\right)}^{n}\right]$$

where w (kg_ref_/kg_PR_ or cm^3^/g_ref_) and wo (kg_ref_/kg_PR_) are the adsorption equilibrium and maximum adsorption uptake possessed by the PR derivatives corresponding to the different refrigerants, T (K) is adsorption temperature, E (kJ/kg) is the characteristic energy, and n is the structural heterogeneity parameter commonly named as D–A fitting constant. P and Ps are the partial and saturation pressures (kPa), respectively. The P_s_ of the refrigerants corresponding to T are obtained from the Coolprop, an open-source Python library for fluid properties. However, beyond the critical temperature (T > T_cr_) of the refrigerants, the P_s_ values are calculated from the pseudo-saturation pressure provided by Equation (2) [41]. In addition, q is evaluated on volumetric bases (cm^3^_ref_/g_PR_) to convert into equilibrium adsorption amount (kg_ref_/kg_PR_) using Equation (3) as provided by [41,42]:

(2) $$P_{s}={\left(\frac{T}{T_{\mathrm{cr}}}\right)}^{k}{\;P}_{c}$$

(3) $$q=\frac{w_{o}}{V_{t}\exp\left(\alpha \left(T-T_{t}\right)\right)}\exp\left(-{\left\{\frac{R_{o}T}{E}\ln\left(\frac{P_{s}}{P}\right)\right\}}^{n}\right)$$

In Equation (2), P_c_ is the critical pressure and k is a fitting constant. The k values for HFC-32 and CO_2_ are provided in Table 3 [41,42]. However, V_t_ is the molar volume of the refrigerant determined by Equation (3) using the Refprop software at triple point temperature (T_t_). The α is the thermal expansion coefficient taken as 0.0025 K^−1^. The V_t_ value of HFC-32 and CO_2_ refrigerants are computed as 0.69966 cm^3^/g_ref_ and 0.84858 cm^3^/gref, respectively.

### 3.2. System Governing Equations

In this section, steady-state governing heat and mass balance equations used in ongoing research are presented and discussed accordingly in order to develop a Python program that is employed to evaluate the performance of the AHP system. A generalized programming code is written in Python for thermodynamic modeling of the AHP system, which is capable of investigating different adsorbent/refrigerant pairs. The scope of the work in this case, however, is confined to analyzing the PR derivative/refrigerant pairs. Clausius–Clapeyron model is utilized for determining the heat of adsorption/desorption (Q_st_), provided by Equation (4) [41]. Accordingly, isosteric heating temperature (T_iso_heating_) and isosteric cooling temperature (T_iso_cooling_) were estimated.

(4) $$\frac{Q_{\mathrm{st}}}{R_{o}}=-{\left[\frac{\partial \ln\left(P\right)}{\partial \;\left(\frac{1}{T}\right)}\right]}_{w=\mathrm{constant}}$$

Once isosteric temperature points are identified, the Python program developed pressure–temperature–uptake (P–T–U) diagram. Four thermodynamic processes, namely (i) isobaric adsorption, (ii) isosteric heating, (iii) isobaric desorption, and (iv) isosteric cooling processes, perform in sequence to complete a single AHP cycle. The time required to complete a single AHP cycle depends upon the adsorption kinetics. However, the primary objective of the present study is to undertake a comprehensive investigation into steady-state phenomena. It is noteworthy that, within the scope of this research, the kinetics pertaining to PR derivative/refrigerant pairs have deliberately been excluded from consideration. Thermodynamic processes involved in the AHP cycle are provided by Equations (5)–(8) [43,47].

Isobaric adsorption (process 1-2);

(5) $$-Q_{1\text{-}2}=\int_{T_{1}}^{T_{2}}(m_{PR}\;cp_{PRd}+w\;m_{PR}\;cp_{ref@T})dT+m_{PRd}\;\int_{w_{min}}^{w_{max}}Qst_{w}\;dw$$

Isosteric heating (process 2-3);

(6) $$+Q_{2\text{-}3}=\int_{T_{2}}^{T_{3}}m_{PR}\;cp_{PRd}\;dT+w_{max}\;cp_{ref@T}\;\int_{T_{2}}^{T_{3}}dT$$

Isobaric desorption (process 3-4);

(7) $$+Q_{3\text{-}4}=\int_{T_{3}}^{T_{4}}(m_{PR}\;cp_{PRd}+w\;m_{PR}\;cp_{ref@T})dT+m_{PRd}\;\int_{w_{max}}^{w_{min}}Qst_{w}\;dw$$

Isosteric cooling (process 4-1);

(8) $$-Q_{1\text{-}4}=\int_{T_{4}}^{T_{1}}m_{PR}\;cp_{PRd}\;dT+w_{min}\;cp_{ref@T}\;\int_{T_{4}}^{T_{1}}dT\left(T_{1}-T_{4}\right)$$

The first term in Equation (5) computes the sensible heat release during the cooling of PR derivative, whereas the second term computes the latent heat release during the adsorption of refrigerant vapors. The total energy release during isobaric adsorption (Q_1-2_) is indicated with a negative sign. The total isosteric heat (Q_2-3_) supplied for increasing the temperature from T_2_ to T_3_ is computed by Equation (6), which is the summation of the sensible heat gained by the PR derivative adsorbent and heat gained by the refrigerant vapors under equilibrium conditions. The desorption heat is supplied from the external low-grade heat source. The heat supplied during isobaric desorption (Q_3-4_) can be computed from Equation (7). The first term calculates the sensible heat supplied to the PR derivative adsorbent for reaching regeneration temperature (T_4_), whereas the second term is the latent heat gained by refrigerant vapors for desorbing from the adsorbent surface. Again, the heat is released during the isosteric cooling process (Q_1-4_) estimated by Equation (8) that is the summation of the two sensible heats for lowering the temperature of PR derivative as well as the refrigerant vapors that still adsorb on the surface. The key performance indicators for both adsorption cooling and adsorption heating are estimated using Equations (9)–(13) as provided by [43,47]:

Evaporator/specific cooling energy (SCE):

(9) $$Q_{\mathrm{evap}}\mathrm{or}\;\mathrm{SCE}=m_{\mathrm{PRd}}\left(w_{\max}-w_{\min@T,P}\right){\;\mathrm{LH}}_{\mathrm{vap}@T_{e}}\;-m_{\mathrm{PRd}}\left(w_{\max}-w_{\min}\right){\mathrm{cp}}_{{\mathrm{ref}}_{l}@T}\int_{T_{\mathrm{evap}}}^{T_{\mathrm{cond}}}\mathrm{dT}$$

Condenser;

(10) $$Q_{\mathrm{cond}}=\left(w_{\max}-w_{\min}\right){\;m}_{\mathrm{PRd}}{\;\mathrm{LH}}_{\mathrm{vap}@T_{\mathrm{cond}}}$$

Specific heating energy (SHE);

(11) $$\mathrm{SHE}={\;Q}_{\mathrm{cond}}+Q_{1\text{-}2}+Q_{1\text{-}4}$$

Coefficient of performance for cooling application (COP_cooling_);

(12) $${\mathrm{COP}}_{\mathrm{cooling}}=\frac{\mathrm{SCE}}{Q_{2\text{-}3}+Q_{3\text{-}4}}$$

Coefficient of performance for heating application (COP_heating_);

(13) $${\mathrm{COP}}_{\mathrm{heating}}=\frac{\mathrm{SHE}}{Q_{2\text{-}3}+Q_{3\text{-}4}}$$

The specific cooling energy (SCE) or Q_evap_ is obtained during the isobaric adsorption process when the refrigerant in the evaporator changes from liquid to vapors by gaining the surrounding heat. In order to compute the refrigeration effect, Equation (9) is utilized, which consists of latent heat of evaporation of the refrigerant minus the sensible heat of refrigerant, which enters into the evaporator at condenser temperature. Similarly, the specific heating energy (SHE) obtained from the single AHP cycle is the function of heat released during condensation plus the heat released during the isosteric cooling and isobaric adsorption. The condenser (Q_cond_) is computed by the latent heat of vaporization at condenser temperature, as provided in Equation (10). The total SHE is calculated from Equation (11). The COP for both cooling and heating is provided by Equations (12) and (13), respectively.

## 4. Results and Discussion

**Figure 3:**
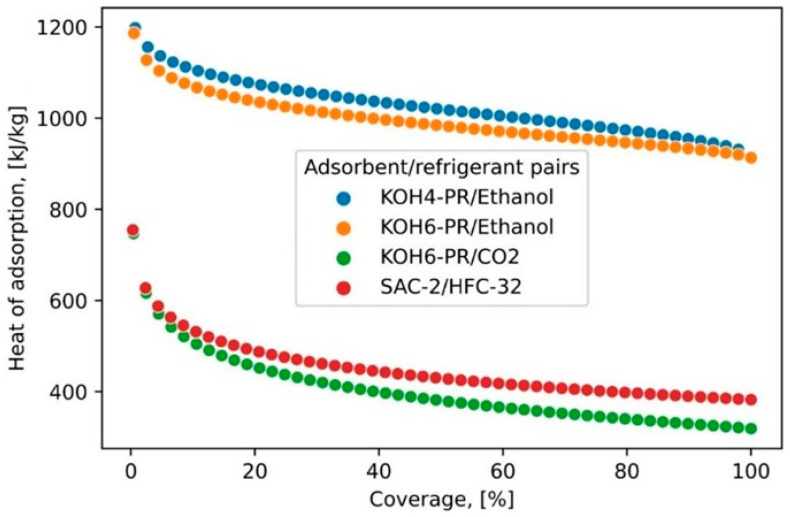
Isosteric heat of adsorption of PR derivative/refrigerant pairs.

Figure 3 shows the isosteric heat of adsorption of PR derivative/refrigerant pairs and the isosteric heat of adsorption (Q_st_) profiles computed for all PR derivative/refrigerant pairs corresponding to the percentage coverage of refrigerants on the adsorbent surface. The Q_st_ values are determined using the Clausius–Clapeyron equation in conjunction with the adsorption equilibrium model. It has been realized that KOH4-PR/ethanol and KOH6-PR/ethanol pairs possess high Q_st_ if compared with KOH6-PR/CO_2_ and SAC-2/HFC-32 pairs. The Q_st_ values in the case of the KOH4-PR/ethanol pair and KOH6-PR/ethanol pair are measured at 1198.0 kJ/kg and 1120.0 kJ/kg at 1% coverage, which declined to 915.71 kJ/kg and 912.93 kJ/kg at 100% coverage (saturation), respectively. For KOH6-PR/CO_2_ and SAC-2/HFC-32, the Q_st_ values vary from 713.70 to 304.85 kJ/kg and 757.49 to 385.09 kJ/kg, respectively. The computed Q_st_ values for each PR derivative/refrigerant pair have been utilized for determining the T_iso_heating_ and T_iso_cooling_ that are later utilized for the development of P–T–U diagrams.

### 4.1. KOH6-PR/CO_2_

**Figure 4:**
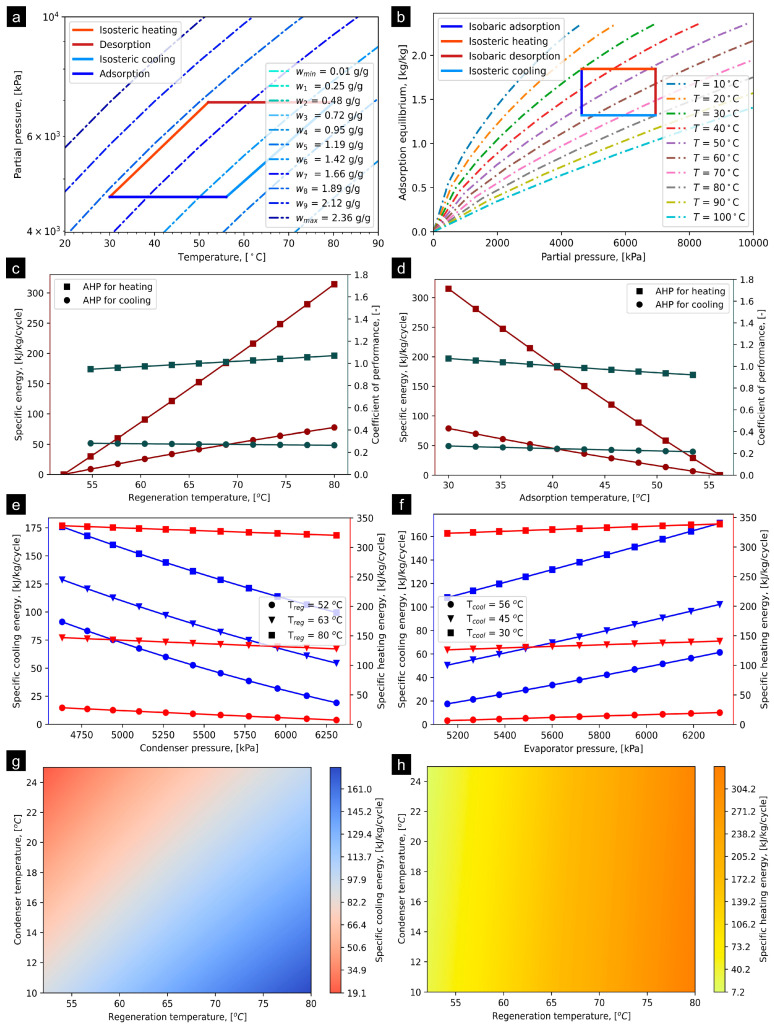
(**a**) P–T–U diagram of KOH6-PR/CO_2_ pair at T_evap_ = 10 °C and T_cond_ = 30 °C, (**b**) simulated adsorption equilibrium profiles entailing ideal AHP cycle, (**c**) effect of T_reg_ on SCE and COP, (**d**) effect of T_ads_ on SCE and COP, (**e**) effect of condenser pressure on SCE and SHE, (**f**) effect of evaporator pressure on SCE and SHE, (**g**) SCE production corresponding to variation in T_reg_ and T_cond_ at T_evap_ = 10 °C, and (**h**) SHE production corresponding to varying T_reg_ and T_cond_ at T_evap_ = 10 °C.

The ideal cycle and/or P–T–U for KOH6-PR/CO_2_ is presented in Figure 4a, determined at operating conditions provided in Table 1. The T_iso_heating_ and T_iso_cooling_ were computed at 54.90 °C and 52.95 °C, respectively. The net adsorption uptake of ethanol on KOH6-PR is estimated to be 0.525 kg/kg at the operational states provided in Table 1. Figure 4b presents the simulated adsorption equilibrium profiles of KOH6-PR/CO_2_ at the temperature range from 10 °C to 100 °C regarding superimposing the AHP cycle. The reference study [41] experimentally investigated the adsorption equilibrium profiles at temperatures of 20 to 70 °C with an interval of 5 °C, and 10 °C, and estimated the fitting constants for the DA model. However, in the present study, the same fitting constants were utilized to simulate the adsorption isotherms at temperatures less than and greater than 20 °C and 70 °C, respectively. The P_evap_ and P_cond_ are assumed to be 4629.83 kPa and 6941.10 kPa, respectively, based on the saturation pressure of CO_2_. Figure 4c shows the effect of T_reg_ from 50 °C to 80 °C on specific energies and COPs that can be obtained from the KOH6-PR/CO_2_ pair. It has been observed that, corresponding to an increase in T_reg_, the SCE and SHE linearly increase. At T_reg_ of 80 °C, the SCE and SHE approach to a maximum potential with a magnitude of 77.44 and 314.58 KJ/kg/cycle. In accordance with increasing the T_reg_ from 51 to 80 °C, the COP for AHP for heating application varies from 0.95 to 1.07, which depicts an increasing trend. However, regarding AHP for cooling application, it has been identified that the COP drops from 0.28 to 0.26. On the other hand, corresponding to an increase in T_ads_, specific energy and COP both for heating and cooling applications are linearly decreased, as shown in Figure 4d. The SCE and SHE were estimated at 88 and 320 kJ/kg/cycle, respectively. Similarly, at T_ads_ of 30 °C, the COP_heating_ was estimated at 4.0-fold higher as compared to the COP_cooling_. Figure 4e shows the effect of P_cond_ on both SCE and SHE at T_reg_ of 52 °C, 63 °C, and 80 °C, differentiated with markers. In the case of the SCE, the P_cond_ was observed to be sensitive to both P_cond_ and T_reg_. However, for SHE, relatively small variation is observed corresponding to an increase in the P_cond_, while there was significant improvement with respect to the step-based increment in T_reg_. This implies that the influence of the P_cond_ is unremarkable on SHE for the KOH6-PR/CO_2_ pair. The maximum SCE of 175 kJ/kg/cycle and SHE of 340 kJ/kg/cycle are observed at P_cond_ and T_reg_ of 4525 kPa and 80 °C, respectively. Similarly, the SCE and SHE are observed to be sensitive and insensitive to P_evap_, respectively, as shown in Figure 4f. However, T_cool_ significantly enhances the magnitude of both SCE and SHE. The maximum SCE of 165.40 kJ/kg/cycle and SHE of 340 kJ/kg/cycle were observed at P_evap_ = 6360 kPa and T_cool_ of 30 °C. Figure 4g,h show the detailed impact of T_cond_, varying from 10 to 25 °C, and T_reg_ from 51 to 80 °C at T_evap_ = 10 °C on both SCE and SHE for developing better understanding as well as visualization of the trends. It can be concluded that the impact of T_cond_ is highly sensitive for SCE, whilst T_cond_ does not significantly impact the SHE at T_reg_ > 75 °C. The extended results regarding the influence of operating parameters on the SCE, SHE, COP_cooling_, and COP_heating_ are provided in Appendix A accordingly.

### 4.2. SAC-2/HFC-32

**Figure 5:**
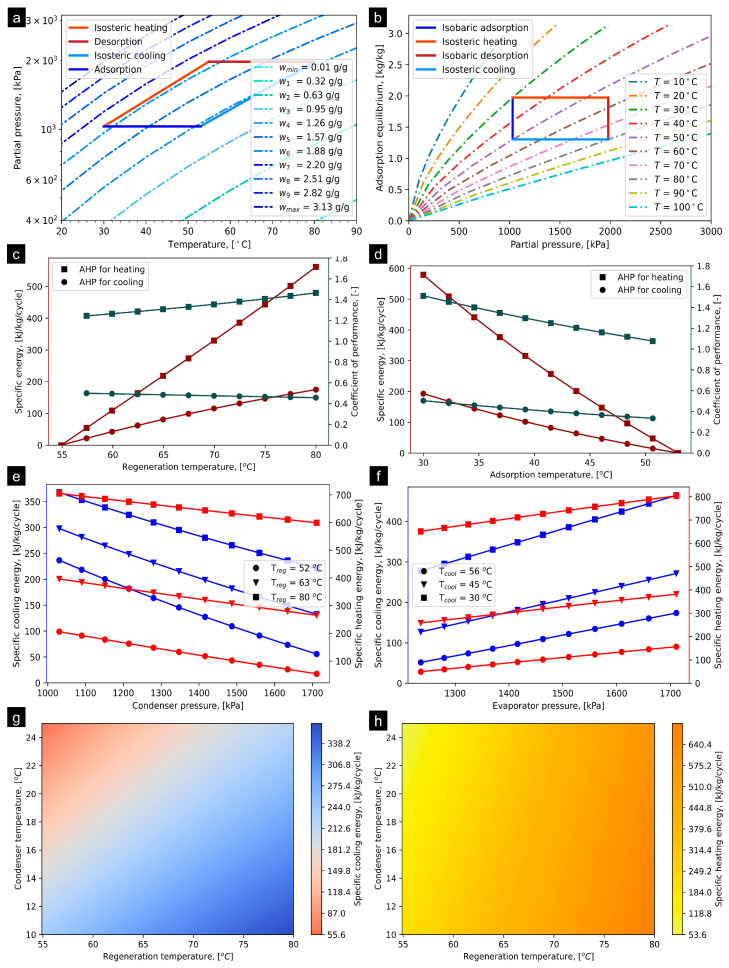
(**a**) P–T–U diagram of SAC/HFC-32 pair at T_evap_ = 10 °C and T_cond_ = 30 °C, (**b**) simulated adsorption equilibrium profiles entailing ideal AHP cycle, (**c**) effect of T_reg_ on SCE and COP, (**d**) effect of T_cool_ on SCE and COP, (**e**) effect of condenser pressure on SCE and SHE, (**f**) effect of evaporator pressure on SCE and SHE, (**g**) SCE production corresponding to variation in T_reg_ and T_cond_ at T_evap_ = 10 °C, and (**h**) SHE production corresponding to varying T_reg_ and T_cond_ at T_evap_ = 10 °C.

Figure 5a presents the P–T–U diagram of AHP-packed SAC-2/HFC-32 pairs with the operating conditions mentioned in Table 1. The T_iso_heating_ and T_iso_cooling_ were computed to be 54.90 °C and 52.95 °C, respectively. The net absorption uptake of HFC-32 on SAC-2 adsorbent was estimated to be 0.669 kg/kg. Figure 5b presents the simulated adsorption equilibrium profiles corresponding to variations in adsorption/desorption temperature, superimposed with the ideal AHP cycle. The isobaric adsorption and isobaric desorption processes are taking place at 1030.44 kPa and 1979.49 kPa for P_evap_ and P_cond_, respectively. Figure 5c presents the SCE and SHE obtained from SAC-2/HFC-32. The SCE found 175.20 kJ/kg/cycle, whereas SHE was 561.14 kJ/kg/cycle. Similarly, the COP_cooling_ varied from 0.50 to 0.45, whereas the COP_heating_ was estimated at 1.24 to 1.46, corresponding to the increase in the T_reg_ from 50 to 80 °C. On the other hand, corresponding to the increment in T_ads_ from 30 to 55 °C, the SCE and SHE decrease from 200 to 0 kJ/kg/cycle and from 590 to 0 kJ/kg/cycle, thereby recommending the 30 °C optimum T_ads_ for the AHP system. 

Figure 5e presents the impact of P_cond_ on SCE and SHE while maintaining P_evap_ = 1030.44 kPa. It has been observed that the P_cond_ responds to a linear decrease in SCE and SHE; however, the higher P_evap_ responds to a linear increase in both SCE and SHE, as shown in Figure 5f. It is noteworthy that both P_evap_ and P_cond_ have a significant influence on the performance of the AHP system packed with SAC-2/HFC-32 pair. Figure 5g and (h) show the detailed impact of T_cond_ varying from 10 to 25 °C and T_reg_ from 51 to 80 °C at T_evap_ = 10 °C on both SCE and SHE for developing better understanding as well as visualization of the trends. It can be concluded that the impact of T_cond_ as well as T_reg_ is highly sensitive for both SCE and SHE. The extended results regarding the influence of operating parameters on the SCE, SHE, COP_cooling_, and COP_heating_ are provided in Appendix A accordingly.

### 4.3. KOH4-PR/Ethanol

**Figure 6:**
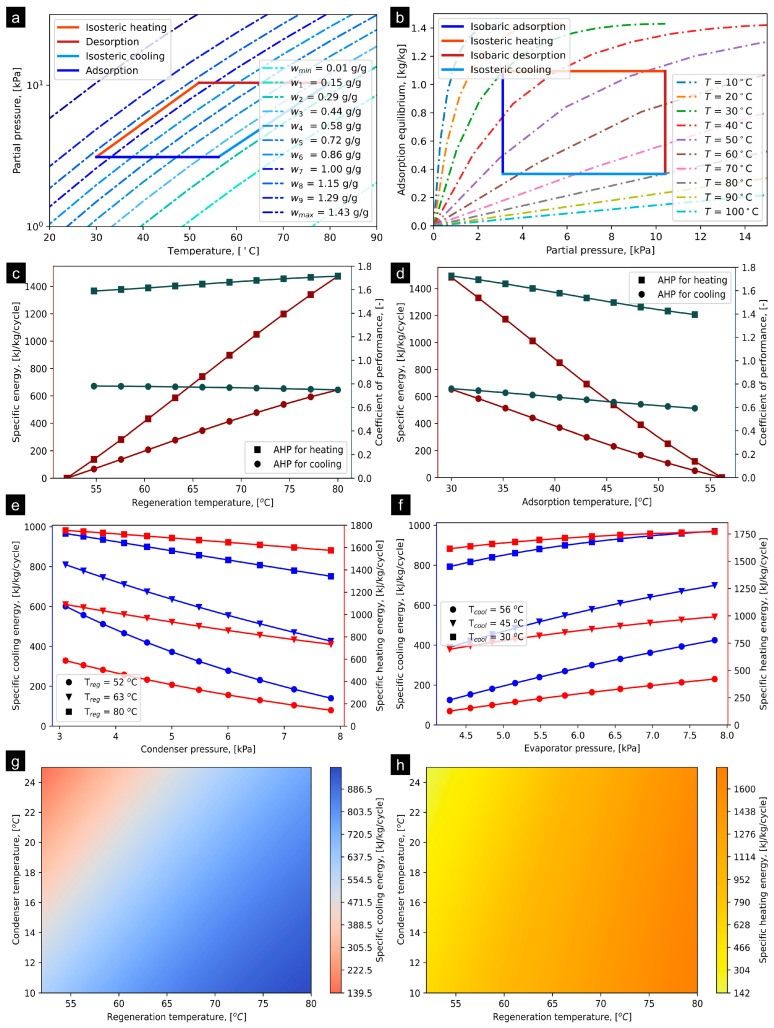
(**a**) P–T–U diagram of KOH4-PR/ethanol pair at T_evap_ = 10 °C and T_cond_ = 30 °C, (**b**) simulated adsorption equilibrium profiles entailing ideal AHP cycle, (**c**) effect of T_reg_ on SCE and COP, (**d**) effect of T_cool_ on SCE and COP, (**e**) effect of condenser pressure on SCE and SHE, (**f**) effect of evaporator pressure on SCE and SHE, (**g**) SCE production corresponding to variation in T_reg_ and T_cond_ at T_evap_ = 10 °C, and (**h**) SHE production corresponding to varying T_reg_ and T_cond_ at T_evap_ = 10 °C.

The ideal cycle and/or P–T–U for KOH4-PR/ethanol is presented in Figure 6a, determined at operating conditions provided in Table 1. The T_iso_heating_ and T_iso_cooling_ were computed to be 51.95 °C and 56.12 °C, respectively. The net adsorption uptake of ethanol on KOH4-PR is estimated to be 0.727 kg/kg at the operational states provided in Table 1. Figure 6b presents the simulated adsorption equilibrium profiles of KOH4-PR/ethanol at a temperature range from 10 °C to 100 °C regarding superimposing the AHP cycle. It is noteworthy that the fitting constants of the adsorption equilibrium were computed for the temperature range between 30 and 70 °C [40]. However, for higher temperatures (>70 °C) and lower temperatures (<30 °C), the adsorption uptakes are simulated using the D–A model varying the temperature parameter. Based on the saturation properties of the ethanol, the P_evap_ and P_cond_ are assumed to be 3.11 kPa and 10.41 kPa, respectively. Figure 6c shows the effect of T_reg_ from 50 °C to 80 °C on specific energies and COPs that can be obtained from the KOH4-PR/ethanol pair. It has been observed that, corresponding to increase in T_reg_, the SCE and SHE linearly increase. At T_reg_ of 80 °C, the SCE and SHE approach maximum potential, with values of 643.85 and 1474.12 KJ/kg/cycle, entailing maximum COP of 1.78 and 0.79, respectively. On the other hand, it corresponds to an increase in T_ads_, with both specific energy and COP both for heating and cooling applications linearly decreasing, as shown in Figure 6d. This implies that the T_reg_ = 80 °C and T_ads_ = 30 °C are supportive in order to obtain the maximum potential (either cooling or heating) from the KOH4-PR/ethanol. Figure 6e shows the effect of P_cond_ on both SCE and SHE at T_reg_ of 52 °C, 63 °C, and 80 °C, differentiated with markers. The study reveals that P_cond_ of 3.11 kPa and T_reg_ of 80 °C is promising in terms of producing high SCE (980 kJ/kg/cycle) and SHE (1790 KJ/kg/cycle). The increment in the T_reg_ reflects the step-based improvement in both SCE and SHE, whilst the degraded corresponds to an increase in P_cond_. Figure 6f shows the effect of P_evap_ and T_cool_ on SCE and SHE. The results depict that nearly linear increments of SCE and SHE correspond to an increase in P_evap_. Also, the step-based increment reflects the significant improvement in the overall SCE and SHE. The maximum SCE of 960 kJ/kg/cycle and SHE of 1750 kJ/kg/cycle were computed at P_evap_ of 7.85 kPa and T_cool_ of 30 °C. Figure 6g,h show the detailed impact of T_cond_, varying from 10 to 25 °C and T_reg_ from 51 to 80 °C at T_evap_ = 10 °C on both SCE and SHE for developing better understanding as well as visualization of the trends. It can be concluded that the impact of T_cond_ on both SCE and SHE is ignorable at T_reg_ > 70 °C. The extended results regarding the influence of operating parameters on the SCE, SHE, COP_cooling_, and COP_heating_ are provided in Appendix A accordingly.

### 4.4. KOH6-PR/Ethanol

**Figure 7:**
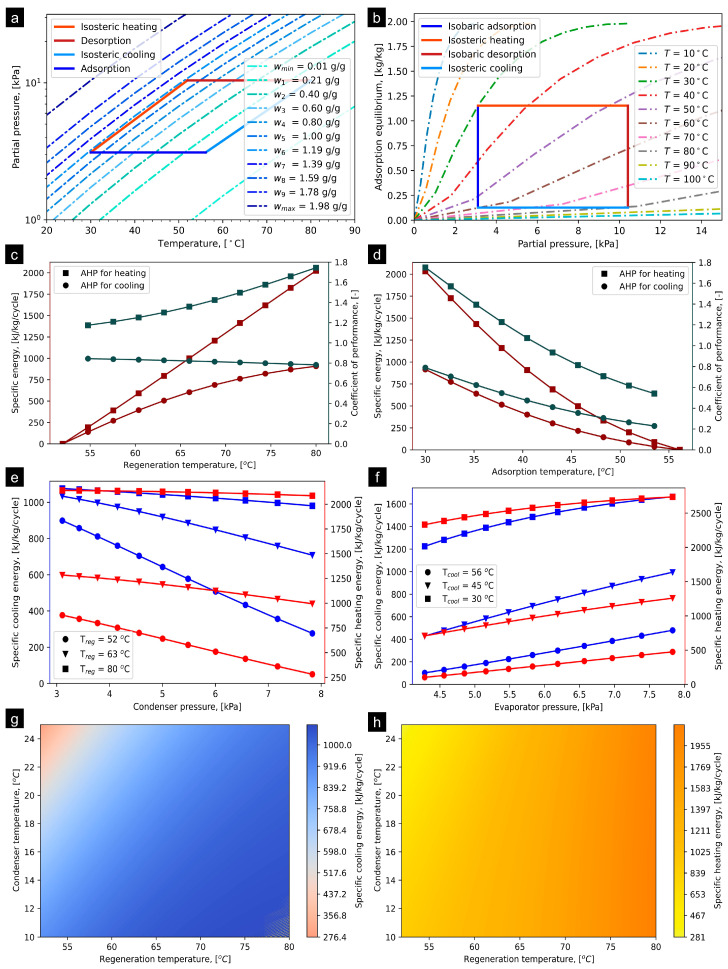
(**a**) P–T–U diagram of KOH6-PR/ethanol pair at T_evap_ = 10 °C and T_cond_ = 30 °C, (**b**) simulated adsorption equilibrium profiles entailing ideal AHP cycle, (**c**) effect of T_reg_ on SCE and COP, (**d**) effect of T_ads_ on SCE and COP, (**e**) effect of condenser pressure on SCE and SHE, (**f**) effect of evaporator pressure on SCE and SHE, (**g**) SCE production corresponding to variation in T_reg_ and T_cond_ at T_evap_ = 10 °C, and (**h**) SHE production corresponding to varying T_reg_ and T_50_ at T_evap_ = 10 °C.

The ideal cycle and/or P–T–U for KOH6-PR/ethanol is presented in Figure 7a, determined at operating conditions provided in Table 1. The T_iso_heating_ and T_iso_cooling_ were computed to be 51.97 °C and 56.10 °C, respectively. The net adsorption uptake of ethanol on KOH6-PR is estimated at 1.0265 kg/kg at the operational states provided in Table 1. Figure 7b presents the simulated adsorption equilibrium profiles of KOH6-PR/ethanol at a temperature range from 10 °C to 100 °C entailing superimposing the AHP cycle. The cited study determined the fitting constants for KOH6-PR/ethanol at a temperature range between 30 and 70 °C [40]; however, in the present study, the adsorption isotherms for higher temperatures (>70 °C) and lower temperatures (<30 °C) are estimated using the D–A model varying the temperature parameter. The P_evap_ and P_cond_ assume 3.11 kPa and 10.41 kPa, respectively. Figure 7c shows the effect of T_reg_ from 50 °C to 80 °C on specific energies and COPs that can be obtained from the KOH6-PR/ethanol pair. It has been observed that, corresponding to an increase in T_reg_, the SCE and SHE linearly increase. At T_reg_ of 80 °C, the SCE and SHE approach to a maximum potential with a magnitude of 907.77 and 2024.34 KJ/kg/cycle, with maximum COP of 0.79 and 1.78, respectively. In the case of AHP for cooling application, the COP followed a declining trend (0.84 to 0.78) due to the mounting of externally supplied heat that leads to a slight drop in the COP. On the other hand, it corresponds to an increase in T_ads_-specific energy and COP both for heating and cooling applications, which follow a relatively curved shape declining trend, as shown in Figure 7d. The SCE and SHE were estimated at 915.00 and 2032.72 kJ/kg/cycle, respectively. Similarly, at T_ads_ of 30 °C, the COP for heating application was estimated 2.34-fold higher as compared to the COP for cooling application. Figure 7e shows the effect of P_cond_ on both SCE and SHE at T_reg_ of 52 °C, 63 °C, and 80 °C, differentiated with markers. The study reveals that P_cond_ of 3.11 kPa and T_reg_ of 80 °C are promising in terms of producing high SCE (1033.78 kJ/kg/cycle) and SHE (2235 KJ/kg/cycle). However, it has been identified that, at T_reg_ = 80 °C, the P_cond_ seems unresponsive to producing SHE for KOH6-PR/ethanol. Similarly, the slope of the SCE profiles corresponds to an increase in the T_reg_ from 52 °C to 80 °C. Figure 7f shows the effect of P_evap_, and T_cool_ on SCE and SHE. The maximum SCE of 1664.21 kJ/kg/cycle and SHE of 2736 kJ/kg/cycle were estimated at P_evap_ of 7.85 kPa and T_cool_ of 30 °C. Figure 7g,h show the detailed impact of T_cond_, varying from 10 to 25 °C and T_reg_ from 51 to 80 °C at T_evap_ = 10 °C on both SCE and SHE for developing better understanding as well as visualization of the trends. It can be concluded that the impact of T_cond_ on both SCE and SHE is ignorable at T_reg_ > 75 °C. The extended results regarding the influence of operating parameters on the SCE, SHE, COP_cooling_, and COP_heating_ are provided in Appendix A accordingly.

## 5. Conclusions

The global transition towards the development of energy-efficient air conditioning systems encourages field researchers to explore the potential of an adsorption heat pump (AHP) system for heat transformation applications. Adsorbent/refrigerant pairs play an important role in the success of the AHP system. In this realm, the study aims to evaluate four kinds of phenol resin (PR) derivative/refrigerant pairs, i.e., (i) KOH6-PR/CO_2_, (ii) SAC-2/HFC, (iii) KOH4-PR/ethanol, and (iv) KOH6-PR/ethanol, for adsorption cooling and adsorption heating applications. A steady-state thermodynamic modeling strategy is utilized containing a Dubinin–Astokhov (D–A) model and governing heat and mass balance equations to explore the performance of the AHP system for the selected PR derivative/refrigerant pairs. Specific cooling energy (SCE), specific heating energy (SHE), and associated coefficient of performances (COP) are estimated in response to influential operating parameters, i.e., regeneration temperature (T_reg_), adsorption temperature (T_ads_), condenser pressure (P_cond_), and evaporator pressure (P_evap_). The analysis reveals that KOH6-PR/ethanol outperforms for the AHP system due to producing the highest SCE of 907.77 kJ/kg/cycle, with SHE of 2024.34 kJ/kg/cycle at T_reg_ of 80 °C, P_cond_ of 10.41 kPa, and P_evap_ of 3.106 kPa, followed by KOH4-PR/ethanol, SAC-2/HFC-32, and KOH6-PR/CO_2_. Accordingly, the COP_cooling_ and COP_heating_ were estimated to be 0.79, and 1.78, respectively. In addition, for KOH6-PR/ethanol, it was observed that condenser temperature (T_cond_) and evaporator temperature (T_evap_) have no and/or relatively less sensitive for higher T_reg_ (>75 °C) and lower T_ads_ (<32 °C), respectively. However, prior to implementation, meticulous consideration of the adsorption kinetics is highly imperative. Therefore, the performance of the KOH6-PR/ethanol pair may exhibit considerable variability, necessitating in-depth exploration and comprehensive analysis of the underlying adsorption kinetics. Conducting further investigations in this domain is crucial to attain a thorough understanding and optimize the overall efficiency of this intricate system.

## Figures and Tables

**Table 1:** Steady-state operating conditions of AHP system.

Parameter/Symbol	Value	Units
Adsorption temperature (T_ads_)	30	°C
Regeneration temperature (T_reg_)	80	°C
Evaporator temperature (T_evap_)	10	°C
Condenser temperature (T_cond_)	30	°C
Cooling bath temperature (T_cool_)	T_ads_-T_iso_cooling_	°C
Hot bath temperature (T_hw_)	T_iso_heating_-T_reg_	°C

**Table 2:** Porous, elemental composition, and thermal properties of the studied PR derivatives, reproduced with permission from [40,41,42].

Parameters	KOH4-PR	KOH6-PR	SAC-2
Porous Properties			
Total surface area (m^2^/g)	3060	2910	2992
Total pore volume (cm^3^/g)	1.90	2.53	2.52
Micropore volume (cm^3^/g)	1.85	2.37	2.29
Pore width (nm)	1.25	1.78	1.62
**Elemental Composition**			
C (-)	0.9537	0.9272	0.9518
H (-)	0.0005	0.0010	0.0022
N (-)	0.0018	0.0022	0.0026
O_diff_ (-)	0.0440	0.0543	0.0434
O/C (-)	0.035	0.044	N/A
Ash (-)	N/A	0.0153	N/A
**Thermal Property**			
Specific heat capacity (kJ/kg.K)	N/A	0.751–0.924 (@ 30–150 °C) [45]	N/A

**Table 3:** The D–A constants for the studied PR derivative/refrigerant pairs, reproduced with permission from [40,41,42].

PR Derivative/Refrigerant Pairs	w_o_ (kg_ref_/kg_PR_)	E (kJ/kg)	n (-)	k (-)	Ref.
KOH6-PR/CO_2_	2.36 *	86.74	1.064	4.799	[41]
SAC-2/HFC-32	3.13 *	67.25	1.0217	3.65	[42]
KOH4-PR/ethanol	1.43	128	2	-	[40]
KOH6-PR/ethanol	1.98	90	1.5	-	

Note: measures of volumetric bases (cm^3^_ref_/g_PR_).

**Figure A1:**
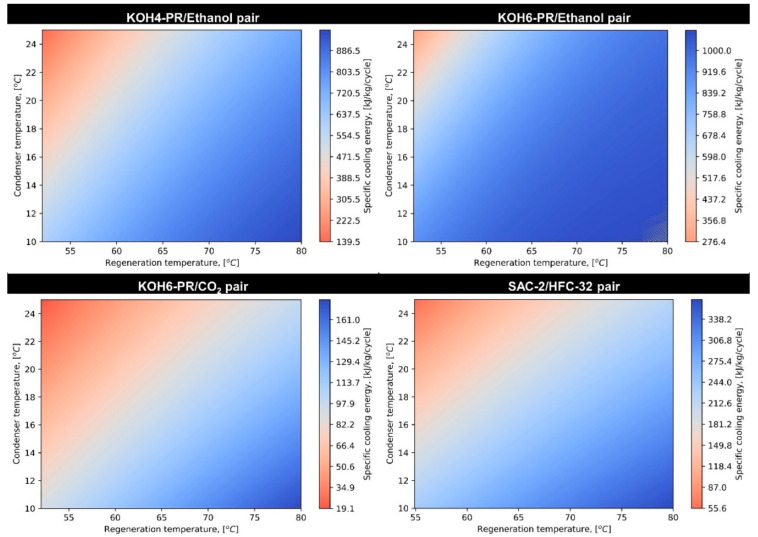
Comparison of PR derivative/refrigerant pairs focusing on determining the effect of T_reg_ and T_cond_ on SCE at T_evap_ = 10 °C.

**Figure A2:**
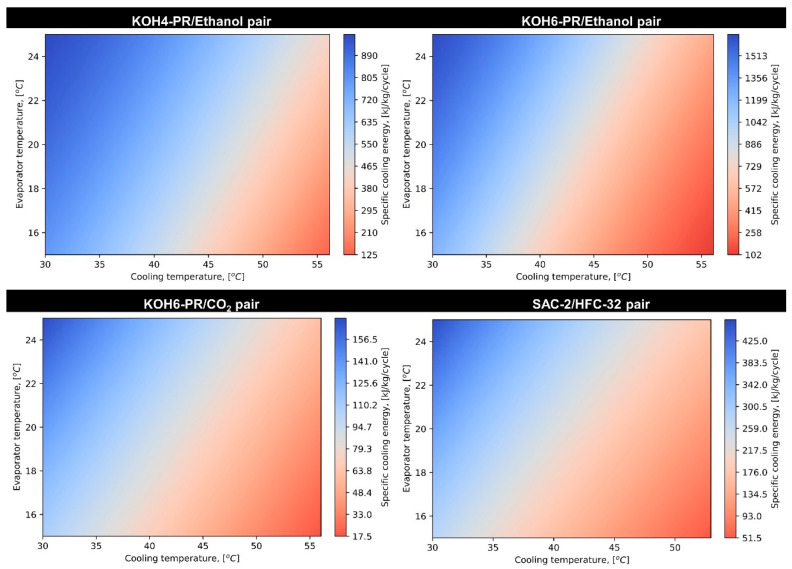
Comparison of PR derivative/refrigerant pairs focusing on determining the effect of T_cool_ and T_evap_ on SCE at T_cond_ = 30 °C.

**Figure A3:**
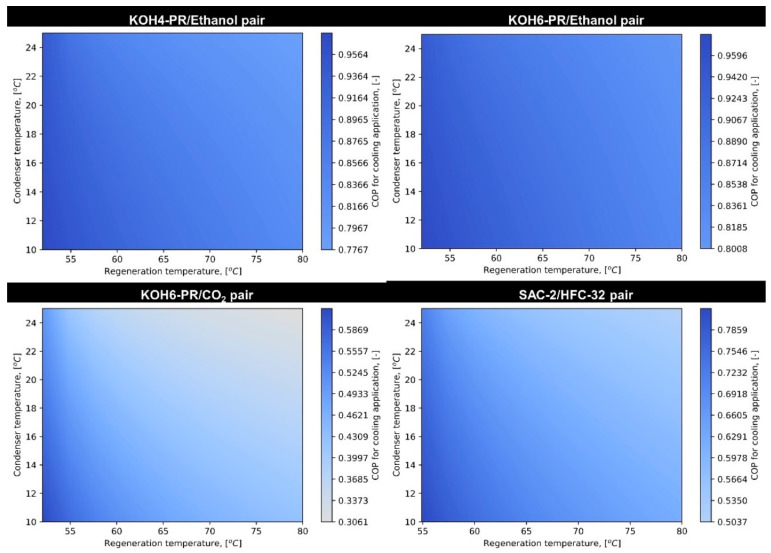
Comparison of PR derivative/refrigerant pairs focusing on determining the effect of T_reg_ and T_cond_ on COP_cooling_ at T_evap_ =10 °C.

**Figure A4:**
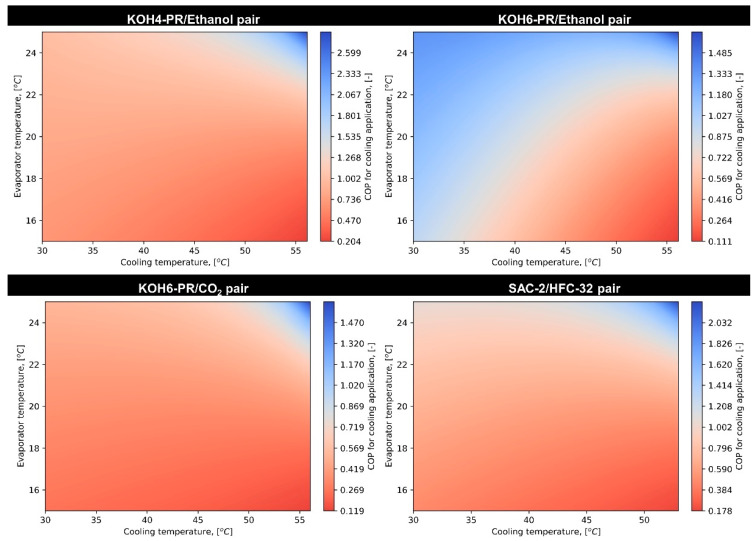
Comparison of PR derivative/refrigerant pairs focusing on determining the effect of T_cool_ and T_evap_ on COP_cooling_ at T_cond_ = 30 °C.

**Figure A5:**
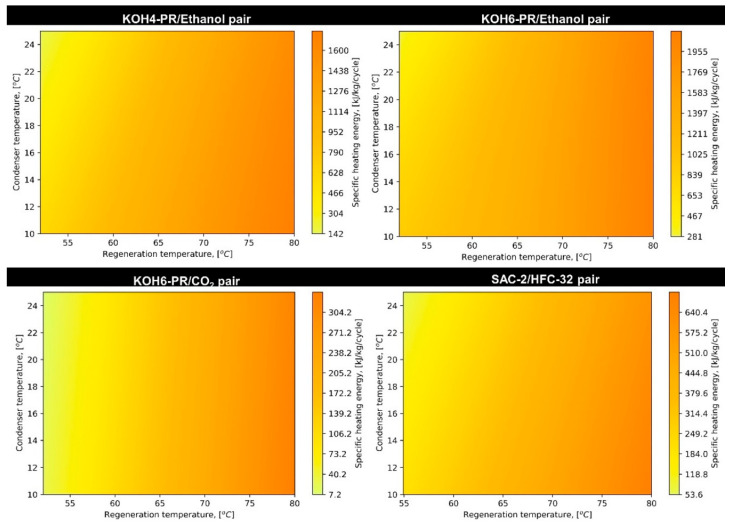
Comparison of PR derivative/refrigerant pairs focusing on determining the effect of T_reg_ and T_cond_ on SHE at T_evap_ = 10 °C.

**Figure A6:**
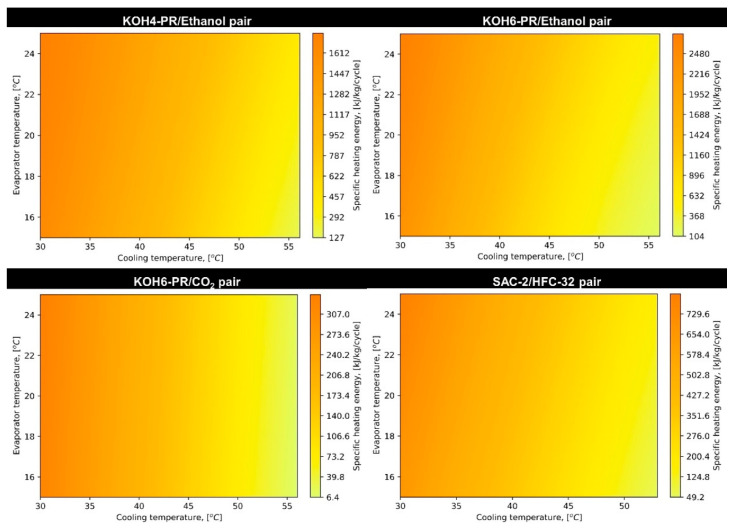
Comparison of PR derivative/refrigerant pairs focusing on determining the effect of T_cool_ and T_evap_ on SHE at T_cond_ = 30 °C.

**Figure A7:**
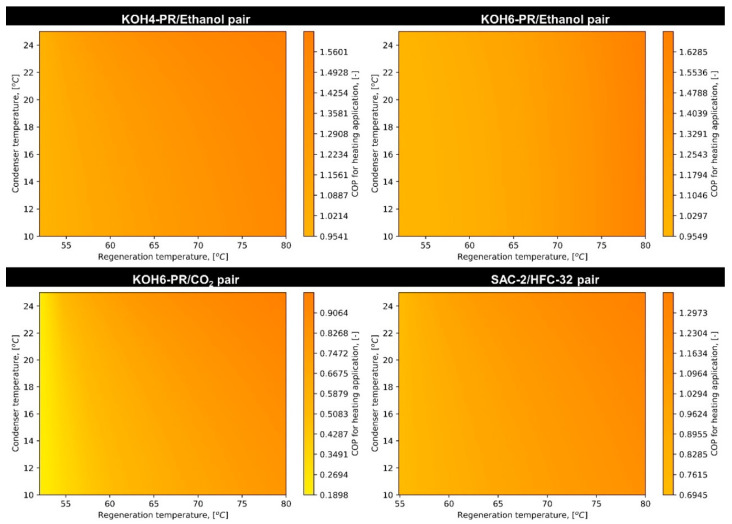
Comparison of PR derivative/refrigerant pairs focusing on determining the effect of T_reg_ and T_cond_ on COP_heating_ at T_evap_ = 10 °C.

**Figure A8:**
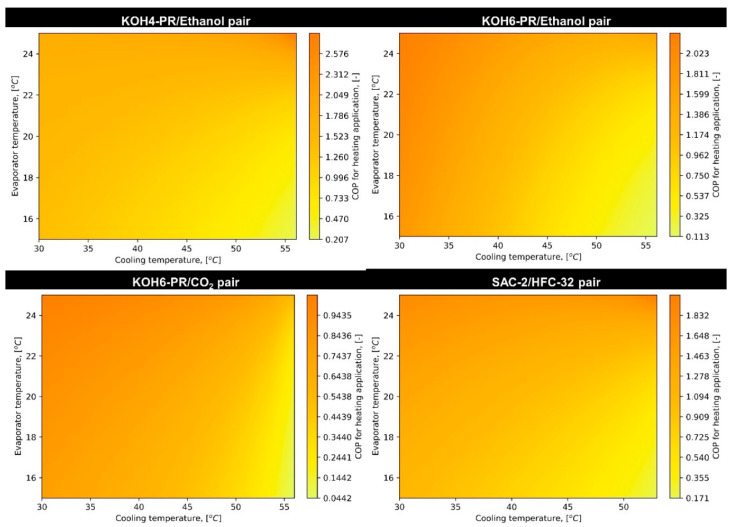
Comparison of PR derivative/refrigerant pairs focusing on determining the effect of T_cool_ and T_evap_ on COP_heating_ at T_cond_ = 30 °C.

## Data Availability

The data are available within the article.

## Appendix A

### Appendix A.1. AHP for Cooling

Figure A1 presents the effect of T_reg_ and T_cond_ on SCE for the selected PR derivative/refrigerant pairs while maintaining the T_evap_ = 10 °C. It has been observed that KOH6-PR/ethanol pair possesses the highest SCE among the selected PR derivative/refrigerant pairs. The SCE value ranges between 276 and 1080 kJ/kg/cycle, followed by KOH4-PR/ethanol pair (139.5–969.5 kJ/kg/cycle), SAC-2/HFC-32 pair (55.60–369.6 kJ/kg/cycle), and KOH6-PR/CO_2_ (19.80–173.56 kJ/kg/cycle), respectively, while varying the T_reg_ from 51 °C to 80 °C and T_cond_ from 10 °C to 25 °C. In Figure A2, the effect of T_cool_ and T_evap_ on SCE is depicted considering T_cond_ = 30 °C. The SCE of KOH6-PR/ethanol estimated >513.0 kJ/kg/cycle at T_evap_ = 25 °C and T_cool_ = 30 °C, followed by KOH4-PR/ethanol (>890 kJ/kg cycle), SAC-2/HFC-32 (>425.0 kJ/kg cycle), and KOH6-PR/CO_2_ (>158.0 kJ/kg cycle). It can be concluded that KOH6-PR/ethanol is superior from the perspective of producing SCE. Figure A3 shows the variation in the COP_cooling_ corresponding to T_reg_ and T_cond_ while maintaining T_evap_ = 10 °C. The COP_cooling_ observed sensitive for both KOH6-PR/CO_2_ and SAC-2/HFC-32 whilst not sensitive for KOH4-PR/ethanol and KOH6-PR/ethanol. Figure A4 presents the COP_cooling_. Similarly, higher T_cool_ placed an adverse impact on the COP_cooling_, as presented in Figure A4. It has been observed that, for KOH4-PR/ethanol, COP_cooling_ value of > 2.59 is estimated followed by KOH6-PR/ethanol (>1.485), SAC-2/HFC-32 (>2.03), and KOH6-PR/CO_2_ pairs, respectively.

### Appendix A.2. AHP for Heating

Figure A5 presents the SHE that might obtained from an AHP system packed with PR derivative/refrigerant pairs. According to the obtained results, KOH6-PR/ethanol possess highest SHE potential, having value of > 1955 kJ/kg/cycle at T_reg_ = 80 °C while irrespective of the T_cond_. Figure A6 shows the effect of T_cool_ and T_evap_ on SHE. It has been realized that maximum of 2744 kJ/kg/cycle of SHE can be harnessed with the AHP system packed with KOH6-PR/ethanol. Figure A7 shows the variation in the COP corresponding to T_reg_, and T_cond_ while maintaining T_evap_ = 10 °C. The COP_heating_ observed sensitive for both KOH6-PR/CO_2_ and SAC-2/HFC-32 whilst not sensitive for KOH4-PR/ethanol and KOH6-PR/ethanol. Beyond the 65 °C of T_reg_, highest COP_heating_ values are observed for both KOH4-PR/ethanol and KOH6-PR/ethanol. Figure A8 presents the COP_heating_ of the AHP system corresponding to varying the T_cool_ and T_evap_. It can be observed that, at low T_cool_ (30–35 °C), maximum SHE is produced, entailing high COP_heating_. The COP_heating_ values for KOH4-PR/ethanol and KOH6-PR/ethanol are observed greater than 2.0, reflecting high heating potential.
